# The role of a moisture-barrier latex in controlling retention, stability and release of D-limonene from complex coacervated matrix microparticles formed during spray drying

**DOI:** 10.3389/fnut.2022.979656

**Published:** 2022-08-25

**Authors:** Yuting Tang, Hayeon Park, Herbert B. Scher, Tina Jeoh

**Affiliations:** Department of Biological and Agricultural Engineering, University of California, Davis, Davis, CA, United States

**Keywords:** complex coacervation, spray drying, latex polymer, volatile oil, controlled release, CoCo process, D-limonene

## Abstract

Limonene from citrus peel oil is valued as fragrance and flavor additives in food and beverages; however, D-limonene is highly volatile and oxygen-sensitive, thus present storage and stability challenges in food products. A novel, industrially-scalable microencapsulation by *in situ* complex coacervation during spray drying process (CoCo process) was applied to encapsulate limonene in alginate-gelatin matrix microparticles. Specifically, we investigated the potential to improve upon prior work demonstrating volatile retention and enteric release of limonene from the complex coacervated (CoCo) microcapsules by incorporating ethylcellulose to improve moisture and oxygen barrier properties of the encapsulation matrix. We hypothesized that ethylcellulose, commonly used as a water-barrier coating with pharmaceuticals, would enhance the ability of CoCo microcapsules to retain and shelf-stabilize limonene. The CoCo process alone could achieve limonene retention of 77.7% ± 1.3% during spray drying, with only ∼10% limonene loss and low oxidation rate after 3-weeks of storage in ambient conditions. Contrary to expectations, incorporating ethylcellulose with the CoCo formulation increased volatile losses of limonene during spray drying and during prolonged storage. Moreover, CoCo powders with ethylcellulose accelerated limonene release in water and simulated gastric fluid, and decelerated release in simulated intestinal fluid—a result that was contrary to targeting enteric release. Instead of simply forming a protective water barrier film in the microparticles during spray drying as envisioned, ethylcellulose appeared to bring limonene to the particle surfaces, thereby enhancing volatile losses, facilitating oxidation and accelerating release in acidic aqueous media. Using ethylcellulose as a model, this study demonstrated the potential to formulate CoCo microparticles using latex excipients to control burst release of the payload followed by long-lasting sustained release in air and in aqueous environments.

## Introduction

Limonene, with a citrus-like smell derived from citrus peel oil, is a valuable renewable byproduct of the citrus industry (1). Limonene is bioactive, with antifungal, bacteriostatic and bactericidal properties that is useful in many applications such as for food packaging and preservation, as an alternative biosolvent in household cleaning products, in cosmetic products including soaps, for medical care, and pest control. (2–6). In the food industry, limonene has been used as a flavoring agent in food products such as chewing gum, citrus juices, vegetables, herbs, candies, beverages and ice-creams (6, 7). In addition, limonene is a potential dietary supplement for its anti-inflammatory and anti-stress properties (8). However, the application of limonene in food products or as nutraceuticals is challenging due to its volatility and its instability to process or environmental stresses such as heat and oxygen. Microencapsulation of limonene can facilitate the application of limonene by prolonging shelf life and controlling release.

Among the different techniques of microencapsulation, complex coacervation is a promising technique to achieve high payloads and controlled release (9). However, conventional complex coacervation is a multi-step process with high cost, making it challenging to scale up (10–12). Previously, we applied an industrially-scalable process to microencapsulate limonene in complex coacervated microparticles formed during spray drying (13–16). The *in situ* complex coacervation process (referred to as the “UC Davis CoCo process” or “CoCo process”) enables the formation of complex coacervated microparticles using a low-cost and industrially scalable spray drying process. In the CoCo process, complex coacervation is prevented prior to spray drying by maintaining the feed pH > pK_*a*_ and pI of the matrix polymers. Upon spray atomization of the feed in the spray-dryer, a volatile base vaporizes from the droplets to decrease the pH such that pK_a, polyuronic acid_ < pH < pI_, protein_, which facilitates electrostatic interactions between oppositely charged matrix polymers to associate by complex coacervation. In combination with electrostatic attractive forces, rapid moisture removal during spray drying exert hydrostatic forces within the drying particles to promote tight associations between the polymers, eliminating the need for chemical crosslinking. In previous studies, we microencapsulated limonene emulsified with gelatin in gelatin-alginate complex coacervated microparticles (referred to as limonene-loaded CoCo powder) with up to ∼80% volatile retention and 16–18% (w/w) limonene loading in the powder (13, 14). The limonene-loaded CoCo powders demonstrated enteric release of limonene, retaining up to 86% of limonene in water, 82% in simulated gastric fluid, and releasing up to 72% of limonene in simulated intestinal fluid (14). In general, higher ratios of gelatin to alginates in the CoCo microparticles favored retention of limonene both in the dry powder and in acidic aqueous media. In another study encapsulating therapeutic peptides by the CoCo process, we found that incorporating a water-barrier latex excipient in the CoCo particles markedly improved the retention of the peptides in water and SGF toward enteric release of the payload (15, 17). While the CoCo process is promising for stabilizing and controlling release of D-limonene, the results from encapsulating the peptides prompted the question of whether water-barrier latexes could improve the robustness of the barrier properties of CoCo microparticles to further limit volatile losses and better control the release of limonene in aqueous media.

Formulation and process development of latex dispersions as film coating on solid substrates for odor and taste masking, improvement of appearance, and protection against environmental conditions have been extensively studied (18–20). A latex dispersion is a colloidal dispersion prepared from any existing thermoplastic water insoluble polymer that forms a thin film as water evaporates if the operational conditions are above the glass transition temperature of the latex polymer (21). The latex polymer ethylcellulose (EC) is an ethyl ether of cellulose that has been widely used in the food and pharmaceutical industries as it is non-toxic, FDA-approved for human consumption, insoluble and impermeable to water (22). A common use of ethylcellulose is for protection against moisture and taste masking. Incorporation of latexes with spray-dried crosslinked alginate microparticles (CLAMs) or CoCo particles has been shown to impact payload release of protein and peptides in aqueous media (16, 23); however, the potential to impact the retention/release of hydrophobic cargo such as limonene remains unexplored.

This study investigated the potential to improve the moisture and oxygen barrier of the CoCo matrix by incorporating ethylcellulose as an excipient. Based on previous observations with hydrophilic payloads, we hypothesized that ethylcellulose should improve the volatile retention, extend shelf-stability and limit release of limonene in acidic aqueous media. Limonene was microencapsulated in the CoCo microparticles using alginate and gelatin as matrix polymers, with and without ethylcellulose to evaluate retention during spray drying and storage, oxidation kinetics during storage, and release kinetics in aqueous media.

## Materials and methods

### Materials

D-limonene, gelatin (type A with an isoelectric point of 7), n-hexane, pepsin, pancreatin 8XUSP were purchased from Sigma Aldrich (St. Louis, MO, United States). High viscosity sodium alginate (GRINDSTED Alginate FD 155) with pKa of 3.5 (Dupont Nutrition and Health, New Century, KS, United States) was selected for maximizing the extent of complex coacervation (16, 24). Succinic acid, ammonium hydroxide, sodium hydroxide, sodium chloride and isopropanol were purchased from Fisher Scientific (Fair Lawn, NJ, United States). Anti-foam reagent was purchased from Spectrum Chemicals Mfg Corp (New Brunswick, NJ, United States). Aquacoat^®^ ECD 30 (aqueous colloidal dispersion of ethylcellulose polymer-30% ethylcellulose stabilized by sodium lauryl sulfate (SLS) and cetyl alcohol) was provided by Colorcon (Harleysville, PA, United States) and tributyl citrate was purchased from TCI America (Portland, OR, United States). Limonene, carvone and limonene oxide standards were purchased from Sigma Aldrich (St. Louis, MO, United States).

### Formation of complex coacervated (CoCo) microcapsules

Limonene loaded microcapsules were prepared as described previously (14). An emulsion of 1.2:1 (w/w) gelatin:limonene with a small amount of anti-foam reagent was prepared by first using an Ultra-Turrax T-18 (IKA Works, Inc., Wilmington, DE, United States) at 12,000 rpm for 2 min then three passes through a high-pressure homogenizer (BEEi Nano DeBEE 30-4 High Pressure Homogenizer) at 20 kpsi. A CoCo solution containing gelatin, alginate and succinic acid was prepared, where ammonium hydroxide was used to adjust the solution pH to 8.5. Tributyl citrate was added to the Aquacoat^®^ ECD 30 dispersion (referred to as EC dispersion) at 25% of the mass of ethylcellulose, and rotated at 20 rpm overnight before adding to the CoCo’ solution. The emulsion and the CoCo solution with or without EC dispersion were mixed to form the spray drying feed. Table 1 shows the composition of each formulation. The feed was spray dried by a Buchi B290 laboratory spray dryer using the following conditions: 150°C inlet air temperature, 35 m^3^/h aspirator airflow rate, 6 ml/min feed peristaltic pump (20% of maximum), and 40 mm nozzle pressure.

**TABLE 1:** Formulation to form limonene-loaded CoCo powders with or without ethylcellulose.

Sample ID	Concentration in spray dryer inlet feed (%, w.b.)					
	Total solid	EC dispersion	Alginate	Gelatin	Succinic acid	Limonene
CoCo	7.00	0	0.5	4	0.75	1.75
Low-EC CoCo	7.67	0.5	0.5	4	0.75	1.92
High-EC CoCo	9.33	1.75	0.5	4	0.75	2.33

### Emulsion size and particle size measurement

D-limonene droplet size in the feed and spray dried particle size were measured at room temperature (∼ 25°C) by light scattering as previously described (Mastersizer 3000, Malvern Instrument, Westborough, MA, United States) (14, 25). For the size measurement of limonene emulsion, material type of limonene was with refractive index 1.47, and dispersant type of water was with dispersant refractive index of 1.33. Spray dried powders were dispersed in propan-2-ol to prevent swelling, then dispersed by sonication. Material type of particle was with refractive index 1.57 and absorption index of 0.01, and dispersant type of propan-2-ol was with refractive index of 1.37. Each sample was measured in triplicate.

### Measurement of volatile retention of D-limonene in CoCo microcapsules

Volatile retention of limonene was expressed as the ratio of total limonene in the microcapsules to total limonene in the spray drying feed (14). Limonene content in the spray drying feed and dried powders were measured as previously described (14). Briefly, limonene in the spray drying feed was measured by extracting 0.5 g of the feed emulsion with 10 ml isopropanol, 10 ml hexane and 9.5 g water, mixing by rotation at 20 rpm for 2 h at room temperature and 5–10 min static incubation for separation. The volume of the top hexane layer containing limonene was measured, then sampled and analyzed by gas chromatography (GC).

Spray-dried powders dispersed in water to 1% (w/v) and pH adjusted with sodium hydroxide to > pI of gelatin was rotated end-over-end at 20 rpm for 2 h, including in 45 °C water bath for 10 min to facilitate the dissolution of gelatin. Once the powders were fully dissolved, limonene was extracted using 6 ml isopropanol and 10 ml hexane and 1 h rotation at 20 rpm. The extract mixtures were centrifuged at 1,000 *g* for 10 min. The volume of the top hexane layer containing limonene was measured, then sampled and analyzed by GC. Each powder sample was extracted in triplicate.

The surface limonene content of limonene-loaded CoCo powders was measured using 0.05 g spray-dried powders in 10 ml hexane rotated at 20 rpm for 10 min. The extract mixtures were centrifuged at 8,000 *g* for 2 min. The supernatant was sampled and analyzed by GC.

### Shelf-life study

**FIGURE 1:**
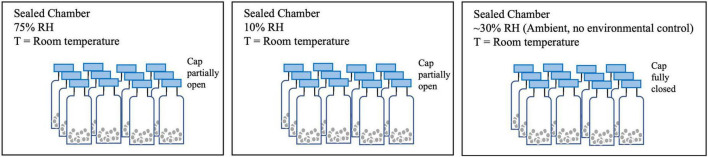
The storage conditions for shelf-life study.

The microcapsules were subjected to three different storage conditions to evaluate the release and oxidation of limonene over time (Figure 1). Storage condition 1 was held at 75% relative humidity (RH) using a saturated NaCl solution (RH 75%). Storage condition 2 was held at < 10% RH using asaturated KOH solution (RH 10%). Storage condition 3 had no environmental controls, with ∼30% RH (RH ∼30%). All three storage conditions were at room temperature (∼22°C).

Powder samples of 0.1 g (for total limonene analysis) or 0.05 g (for surface limonene analysis) were spread in a thin layer in 15-ml glass vials (20 × 48 mm^2^) in triplicates per time point for each storage condition. The glass vials were stored in airtight containers (BD GasPak™) at the three storage conditions. Samples were taken out at each time point (1, 2, and 3 weeks) and the limonene content of the powders was measured as described above. Each powder sample was extracted in triplicates.

### Release of limonene in water, simulated gastric and intestinal fluids

The powders were dispersed in distilled water (pH 5.8), simulated gastric fluid (SGF, 8.78 mg/ml sodium chloride at pH 1.8 with pepsin activity of 2,000 U/ml (26)) and simulated intestinal fluid (SIF, 50 mM phosphate buffer at pH 7.4 with pancreatin activity of 100 TAME U/mL based on trypsin activity (9.6 mg/ml pancreatin 8XUSP)) (14, 25). Specifically, the powders were dispersed in the respective fluids at 0.5% (w/v) in triplicate per time point and incubated at 37 °C (SGF and SIF) or room temperature (water) and rotated end-over-end at 20 rpm. At each time point, 10 mL of hexane was added and mixed very gently for 30 s before centrifuging at 10,000 *g* for 5 min. The supernatant was separated and mixed with 6 ml isopropanol. The mixtures were centrifuged at 1,000 *g* for 10 min after 15 min extraction time. The volume of the top layer containing limonene was measured, then sampled and analyzed by GC. Each powder sample was measured in triplicate.

### Release kinetics

Time course data of limonene release from the CoCo powders in SGF and SIF were fit to a single exponential equation as follows:


(1) $$Q=kt^{n}$$

Where Q is the percentage of limonene release at time t, t is time (min), and k (%/min*^n^*) and n are fitting parameters indicating release rate and release order, respectively.

### Limonene analysis by gas chromatography

The limonene, carvone and limonene oxide concentrations in various extracts were analyzed using a gas chromatograph (Shimadzu 2010) equipped with a DB-FFAP capillary column (30 m × 0.32 mm ID, film thickness 0.25 μm) and flame ionization detector. The injection port and detector temperatures were 250 °C and 260 °C, respectively. The temperature program for each sample started at 50 °C for 3 min, ramped to 190°C at 20 °C/min, held for 1 min, ramped to 200°C at 10°C/min then held for 6 min. Helium was used as the carrier gas. Standard curve of limonene, carvone, and limonene oxide ranged from 7.8 to 2,000 μg/mL, 0.16–20 μg/mL and 0.78–50 μg/mL, respectively.

### Statistical analysis

Experiments were conducted and analyzed in triplicates. Statistical analyses were performed using JMP (SAS Institute Inc., Cary, NC, United States). Independent sample *t*-test was used to determine differences between treatments at selected timepoints. *P*-value less than 0.05 were considered significant.

## Results and discussion

### The impact of ethylcellulose on the volatile retention of limonene in CoCo microcapsules during spray drying

**FIGURE 2:**
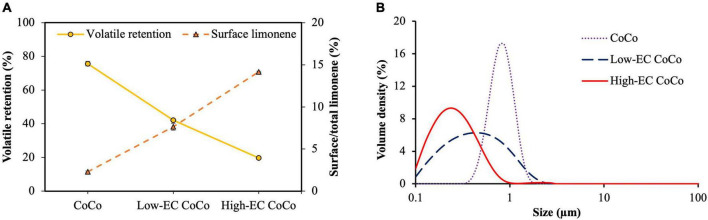
**(A)** Volatile retention and surface content of limonene in CoCo microcapsules after spray drying; **(B)** emulsion size of limonene in the CoCo feed with different concentrations of ethylcellulose. Formulations for CoCo, low-EC CoCo and high-EC CoCo are given in Table 1.

The effect of ethylcellulose in the CoCo microcapsules on the volatile retention of limonene during spray drying was investigated by encapsulating limonene in the complex coacervated powder alone (CoCo), with a small fraction of ethylcellulose (low-EC CoCo) and a high fraction of ethylcellulose (high-EC CoCo). We had hypothesized that ethylcellulose would form a barrier film during particle formation in the spray dryer to improve the retention of limonene within the CoCo matrix. On the contrary, ethylcellulose in the CoCo powder had the unexpected impact of increasing limonene loss during spray drying; the volatile retention of limonene in the CoCo, the low-EC CoCo and the high-EC CoCo were 77.7 ± 1.3%, 42.1 ± 0.5% and 19.7 ± 0.2%, respectively (Figure 2A). Not only did the addition of ethylcellulose reduce volatile retention of limonene during spray drying, higher ethylcellulose content exacerbated volatile losses. Additionally, the surface limonene content increased with higher ethylcellulose content in the CoCo microcapsules (Figure 2A). The coupled trends of increased surface limonene with decreased volatile retention in the CoCo microcapsules suggests that ethylcellulose may have driven limonene to the surface of the matrix. Higher surface limonene content would expedite volatilization of limonene from the particles, resulting in lower volatile retention.

The size of the limonene emulsion in the feed decreased as the ethylcellulose content increased in the formulation (Figure 2B). The ethylcellulose dispersion is stabilized by sodium lauryl sulfate and cetyl alcohol, which could also help to decrease the size of the limonene emulsion. The smaller emulsion size with increased surface area could facilitate the volatilization of limonene. In this study, volatile retention decreased as the mean emulsion size decreased from 0.9 μm to 0.3 μm (Figure 2). One way in which emulsion size can affect volatile losses of limonene is that smaller emulsion sizes have larger specific surface areas of the limonene droplets, thereby increasing the volatilization rate of limonene during drying. While extensively investigated, however, the relationship between volatile retention and emulsion size remains controversial. Chang et al. proposed that larger atomized droplets require more time for outer film formation, resulting in lower volatile retention (27). Soottitantawat et al. reported that increasing emulsion size from 0.65 μm to 2 μm resulted in markedly decreased retention of flavors in different carrier combinations (maltodextrin with Gun Arabic/soybean soluble polysaccharide/HI-CAP 100), suggesting that the reason is the potential shearing of the larger emulsion droplets during atomization to accelerate volatilization (28). The authors also reported that in some cases, flavor retention increased with the increasing emulsion size, suggesting that an optimal emulsion size for maximizing volatile retention depends on the matrix composition and processing conditions.

Within the parameters of this study, incorporating the ethylcellulose suspension with the CoCo formulation likely improved limonene emulsification and preferentially migrated limonene to the droplet surface during drying. We speculate that the resulting increase in the presence of limonene at the surface combined with smaller limonene emulsion size led to lower volatile retention of limonene during spray drying. While outside of the scope of this current study, further parameters to examine include spray drying conditions impacting thermal and atomization properties such as inlet air temperature, feed flow rate, nozzle pressure, and the shape of the spray in the evaporation chamber (spray angle). Further, the influence of ethylcellulose on the glass transition temperature of the CoCo feed impacting particle morphology and matrix barrier properties should also be considered.

### The impact of ethylcellulose on retention and oxidation of limonene in CoCo microcapsules during storage

**FIGURE 3:**
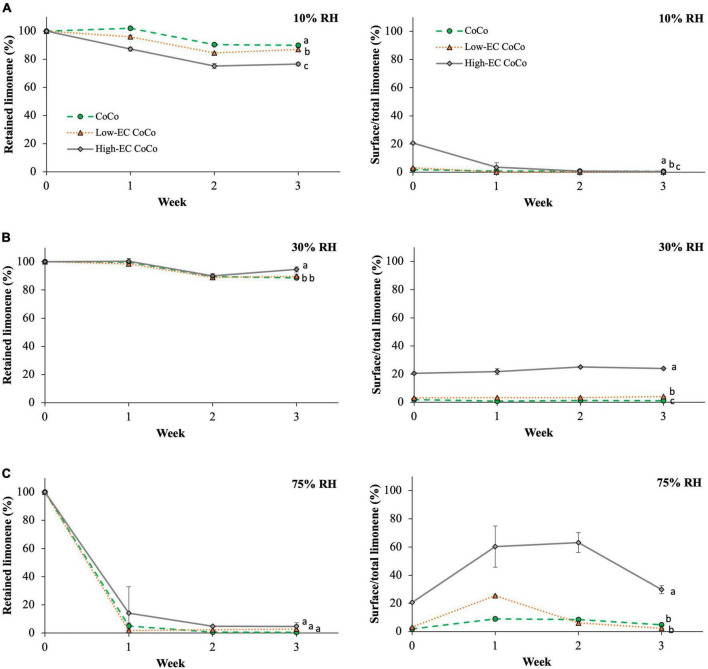
The volatile retention of limonene and the surface limonene content in microcapsules stored at room temperature and **(A)** 10% RH, **(B)** 30% RH, and **(C)** 75% RH. Lines are drawn to guide the eye. Different letters indicate statistically significant differences between different formulations at selected time points.

**FIGURE 4:**
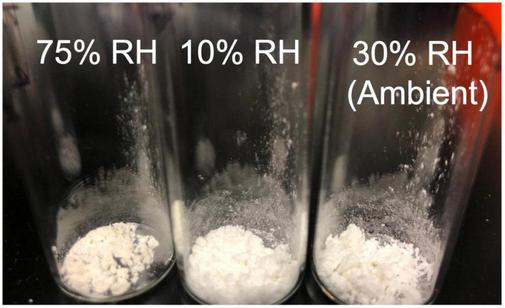
The limonene CoCo powder stored at different conditions after 1 week.

Limonene CoCo microcapsules with varying ethylcellulose contents were stored at 10%, 30% (ambient) and 75% relative humidity to investigate whether ethylcellulose can serve as an effective moisture barrier to aid in retaining limonene during prolonged storage. Relative humidity had a significant effect on volatile retention during storage (Figure 3). Up to 89.9 ± 0.4% and 88.6 ± 0.8% of limonene in the CoCo microcapsules were retained at 10% and ∼30% relative humidity after 3 weeks, whereas limonene retention dropped to 0.6 ± 0.1% at 75% relative humidity (Figure 3). The surface limonene content on CoCo powders increased to 8.9 ± 1.4% after 1 week at 75% relative humidity, compared to less than 1.2 ± 0.0% at lower relative humidity conditions (Figure 3). High relative humidity appeared to facilitate the migration of limonene to the surface of the matrix, resulting in dramatic decreases in limonene contents of the microcapsules. The influence of high relative humidity on volatile retention was consistent with the findings reported by Soottitantawat et al. (29). CoCo powders stored at high relative humidity also exhibited decreased powder quality as evidenced by yellowing and clumping (Figure 4).

Incorporating ethylcellulose in the CoCo microcapsules did not improve the retention of limonene at 10% and 75% RH conditions over the 3 week period (Figure 3). Ethylcellulose in CoCo powders had no influence on limonene content during storage at 75% RH; but ethylcellulose in CoCo increased volatile losses of limonene in the low, 10% relative humidity condition. After 3 weeks at 10% RH, limonene content in high-EC CoCo microcapsules was 76.6 ± 1.3%, significantly lower than 89.9% and 86.9% in the CoCo and the low-EC CoCo microcapsules, respectively (Figure 3A). Compared to CoCo powders, high-EC CoCo powders had increased surface limonene contents at relative high humidity conditions (Figures 3B,C). High-EC CoCo powders also showed decreased surface limonene contents from 0 to 1 week at relative low 10% humidity conditions (Figure 3A), likely because diffusion of limonene through the matrix to the surface is slower than the rate of limonene loss to the atmosphere. Contrary to expectations that ethylcellulose might act as a moisture barrier to slow down to the release of limonene, the data in Figure 3 suggest that ethylcellulose in the CoCo microcapsules facilitated the diffusion of limonene through the matrix to the surface during storage. Moreover, storage at high humidity accelerated the migration of limonene to the surface.

**FIGURE 5:**
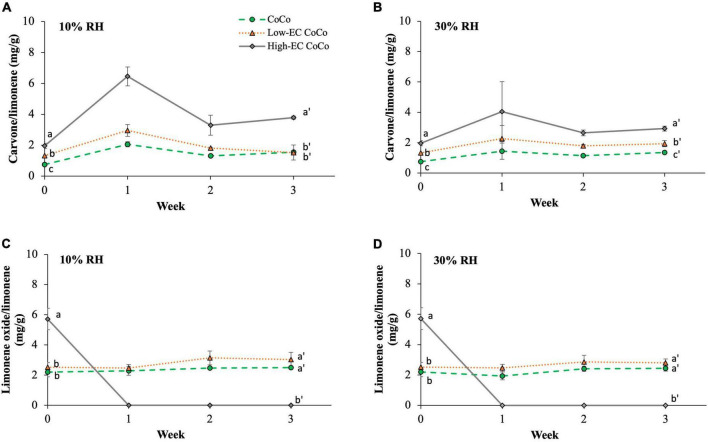
The carvone **(A,B)** and limonene oxide **(C,D)** contents in microcapsules stored at 10% RH condition **(A,C)** and ambient (30% RH) conditions **(B,D)**. The carvone and limonene oxide contents in microcapsules stored at 75% RH is not shown because it was below the detection limits. Lines are drawn to guide the eye. Different letter indicates statistically significant differences between different formulations at selected time points.

Carvone and limonene oxide contents of the powders were measured as indicators of limonene oxidation during storage (Figure 5). CoCo microcapsules with higher ethylcellulose content (high-EC CoCo) saw higher carvone contents (Figures 5A,B) at 10% and 30% RH after 3 weeks. Neither carvone nor limonene oxide were detected in the powders stored at 75% RH, suggesting that the high humidity environment accelerated volatilization of these compounds. Interestingly, the limonene oxide content was the highest in the high-EC CoCo microcapsules immediately after spray drying, but fell below detection within 1 week of storage at either 30% or 10% RH (Figures 5C,D). In contrast, the limonene oxide contents of low-EC CoCo and CoCo powders were relatively steady over the 3 week storage period. The limonene oxide content in the low-EC CoCo microcapsules and the CoCo microcapsules were not significantly different. While the data in Figure 5 are not sufficient to indicate if ethylcellulose in the CoCo powders accelerated limonene oxidation *per se*, the data do suggest that ethylcellulose did not enhance the oxygen barrier of the CoCo microparticles. Moreover, one could speculate that the apparent partitioning of limonene to the surface of the particles by ethylcellulose in the CoCo powders during spray drying (Figure 2) and storage (Figure 3) would in fact facilitate more rapid oxidation of limonene.

### The impact of ethylcellulose on the release of limonene from CoCo microcapsules in aqueous media

**FIGURE 6:**
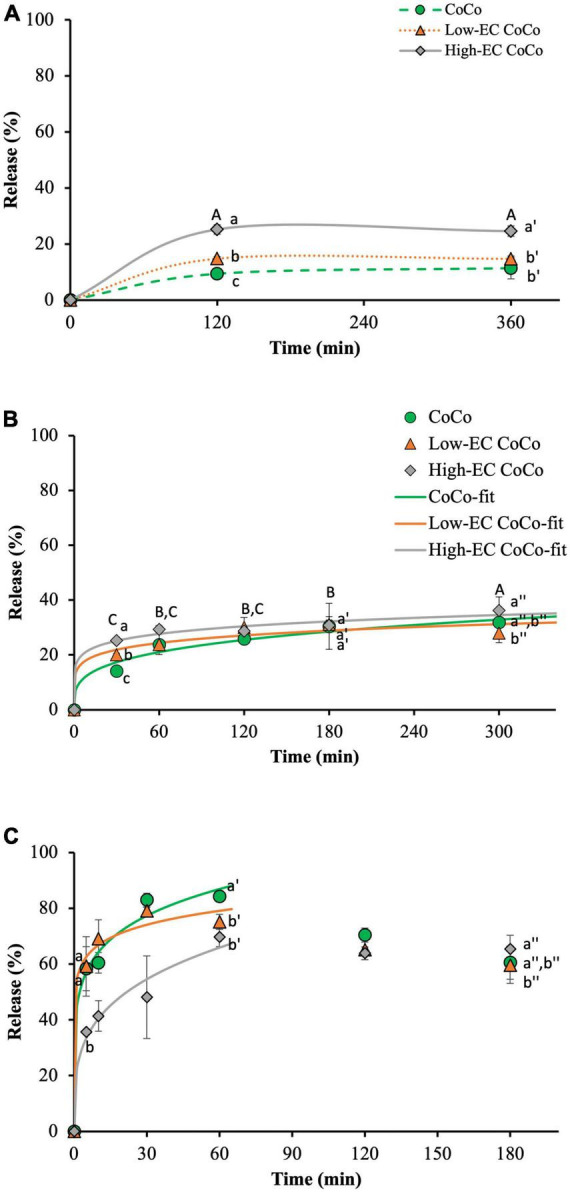
The release of limonene from CoCo, low-EC CoCo and high-EC CoCo microcapsules in water at room temperature **(A)**, SGF at 37°C **(B)** and SIF at 37°C **(B)**. Lines are drawn to guide the eye in panel **(A)**, fits to Equation 1 for the 5 h release of limonene in SGF and the first 1 h release in SIF are shown in panels **(B,C)**. Different letter indicates statistically significant differences between different formulations at selected time points or between different time points in CoCo and low-EC CoCo.

The kinetics of limonene release from the CoCo microcapsules was investigated in water, simulated gastric fluid (SGF) and simulated intestinal fluid (SIF) (Figure 6). Ethylcellulose loading in the CoCo powders affected the release of limonene in the aqueous media. In water, the high-EC CoCo microcapsules released 25.2 ± 1.6% of the encapsulated limonene in 2 h, compared to 14.8 ± 0.7% from the low-EC CoCo microcapsules and 9.4 ± 1.0% from the CoCo microcapsules (Figure 6A). Regardless of the ethylcellulose content, limonene release from the CoCo microcapsules in water was confined within the first 2 h. No further release of limonene was apparent for the next 4 h, demonstrating that the bulk of the limonene in the CoCo microcapsules remained encapsulated for extended storage in water. As noted when assessing volatile retention, it appears that incorporation of ethylcellulose in the CoCo microparticles enhanced the presence of limonene near the particle surfaces, facilitating a burst release in water. Here again, higher amounts of ethylcellulose exacerbated the surface partitioning of limonene to increase initial release.

Faster initial release of limonene from the high-EC CoCo microcapsules was also observed in the SGF. At 30 min, 25.2 ± 1.2% of limonene was released from the high-EC CoCo microcapsules, compared to 20.0 ± 0.6% from the low-EC CoCo microcapsules and 14.1 ± 1.6% from the CoCo microcapsules (Figure 6B). In addition to the partitioning of limonene to the particle surfaces, another possible contributor to accelerated release of limonene in water and SGF from the CoCo microcapsules by ethylcellulose may be enhanced diffusivities of the smaller limonene emulsion droplets through the matrix. Furthermore, our previous studies demonstrated that the primary mechanism of limonene release from CoCo microcapsules is by dissolution of the matrix (14). The potential for dissolution, quantified as the extent of complex coacervation (the ratio of insoluble, coacervated polymers to the total polymers in the matrix) correlated strongly with the release of limonene in water and SGF (14). While the extent of complex coacervation was not measured in this particular study, we speculate that incorporating ethylcellulose in the CoCo microcapsules could interfere with complex coacervation of gelatin and alginate during spray drying, leading to faster matrix dissolution and payload release in acid media. Future work should be carried out to better understand the potential interference of ethylcellulose on complex coacervation between gelatin and alginate. The effect of ethylcellulose on limonene release in SGF was only evident in the early release phase (<1 h). Beyond 1 h incubation, particle composition did not appear to impact limonene release. Further, similar to the release kinetics in water, limonene release in SGF appeared mostly confined to an initial burst release from the powders. There was some indication of slow release of limonene from high-EC CoCo between 1 and 5 h of incubation at 37°C (Figure 6).

In contrast to the effect of ethylcellulose in the CoCo particles on limonene release in water and SGF, the high loading of ethylcellulose in CoCo microcapsules slowed the release of limonene in SIF (Figure 6C). Specifically, up to 58.4 ± 7.9% of limonene was released from CoCo microcapsules in 5 min and increased to 83.0 ± 2.4% in 30 min, whereas only 35.7 ± 0.1% and 48.1 ± 14.8% of limonene released from the high-EC CoCo microcapsules in 5 and 30 min, respectively. The pH of the SIF, pH 7.4, is greater than the pK_*a*_ of gelatin; thus, hydration of the CoCo particles in SIF can disrupt and dissolve the alginate-gelatin matrix to fully release the encapsulated payload. The incorporation of the higher level of ethylcellulose in the CoCo microcapsules attenuated the burst release of limonene in SIF, possibly by interfering with matrix dissolution in the higher pH media. Further studies are needed to understand the precise interactions between ethylcellulose and the matrix polymers to control of the release kinetics of from CoCo microparticles. The current release work in SGF and SIF was not done sequentially, but we speculate that after partial release of limonene in SGF, subsequent incubation in SIF would dissolve the matrix and release the majority of residual limonene from the CoCo powders.

Limonene release in the first 5 h in SGF and the first hour in SIF could be modeled as monotonic increases (Equation 1, Figure 6, and Table 2). As described previously by Siepmann and Peppas (30), release orders of *n* = 0.43 and *n* = 0.85 can be interpreted as diffusion-limited release and swelling-controlled (nearly time-independent) release from spherical particles, respectively. In this study, *n* < 0.5 for all modeled scenarios, indicating that limonene release is likely due to diffusion through the particle matrix, but additional factors further limit release rates. Estimated values of the release rate constant, k, support observations from Figure 6 that limonene release from CoCo with ethylcellulose (Low-EC CoCo and High-EC CoCo) is about twice as fast as CoCo without ethylcellulose (Table 2). Moreover, that high ethylcellulose content in CoCo powders decreased release rates of limonene in SIF (Table 2). High values of k (44%/min from CoCo, Table 2) confirm rapid release of the payload in the intestinal fluid.

**TABLE 2:** Parameter estimates from fitting the release of limonene from CoCo, low-EC CoCo and high-EC CoCo microcapsules in SGF and SIF over time (Figures 6B,C).

Release medium	Paramenters/SSE	CoCo	Low-EC CoCo	High-EC CoCo
SGF	k (%/min^n^)	6.7	12.9	15.8
	n	0.3	0.15	0.14
	SSE^1^	281.9	156.7	97.1
SIF	k (%/min^n^)	43.7	53.8	21.7
	n	0.17	0.09	0.27
	SSE^1^	336.7	509.1	706.4

SSE is the sum of squared errors.

### Physical characterization of CoCo microcapsules with and without ethylcellulose

**FIGURE 7:**
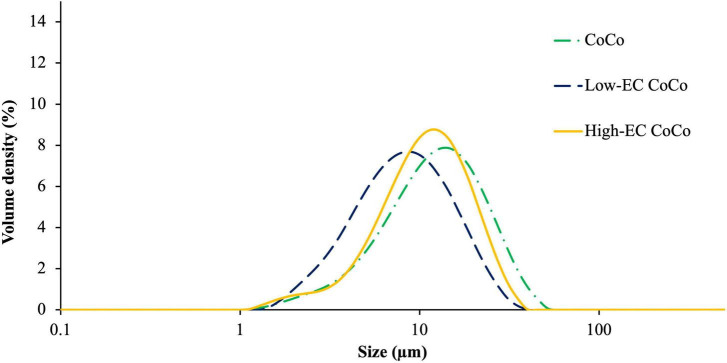
The size distribution of limonene-loaded CoCo, low-EC CoCo, and high-EC CoCo powders.

**FIGURE 8:**
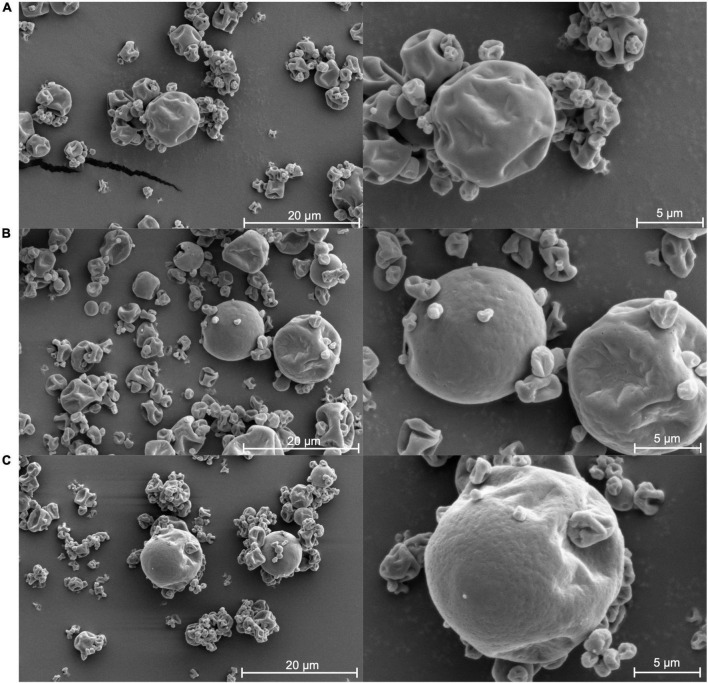
The SEM of microcapsules: CoCo **(A)**, low-EC CoCo **(B)** and high-EC CoCo **(C)**.

The particle size distributions of the CoCo powders formulated with and without ethylcellulose exhibited monomodal size distributions (Figure 7). The volume-weighted mean diameter (D_4,3_), 10th percentile [D(0.1)], median [D(0.5)], and 90th percentile [D(0.9)] of CoCo particles were independent of the ethylcellulose content in the microcapsules. The D_4,3_ of CoCo, low-EC CoCo, and high-EC CoCo microcapsules were 15.1 ± 0.1 μm, 10.2 ± 0.4 μm, and 13.2 ± 0.8 μm, respectively, and they were significantly different from each other. The microcapsules presented with irregular shape with prevalent dents (Figure 8). Larger particles appeared less shrunken compared to smaller particles. Sub-micron sized dimples visible on the surfaces of EC CoCo microcapsules are likely locations of surface limonene droplets that vaporized during imaging. Similar wrinkle features were observed on the surfaces of limonene-oil loaded microcapsules made with maltodextrin and Hi-Cap formed by spray drying (31). The prevalence of such dimples on the surfaces of ethylcellulose containing CoCo microparticles lend support to our conjecture that the higher surface limonene contents by ethylcellulose promoted lower retention of limonene in the microparticles.

## Conclusion

Microencapsulation by *in situ* complex coacervation during spray drying is a potentially economical and commercially-scalable process for stabilizing volatile, hydrophobic payloads such as essential oils and flavors. This work built upon previous success at stabilizing limonene by the CoCo process to study whether incorporation of a latex polymer ethylcellulose with the complex coacervated gelatin-alginate microparticles could improve volatile retention of D-limonene in dry powders and better control release of D-limonene in aqueous media. Contrary to expectations, incorporating ethylcellulose decreased the efficacy of CoCo microparticles at retaining D-limonene during spray drying and storage. Ethylcellulose in CoCo microparticles appeared to promote the migration of D-limonene to the surface of the particles, and the surfactant in the ethylcellulose dispersion decreased the D-limonene emulsion size. Thus, instead of working as a barrier, incorporating ethylcellulose in the CoCo microcapsules increased limonene losses during spray drying and escalated release and oxidation of D-limonene in dry powders during storage, especially at high relative humidity conditions. Moreover, ethylcellulose in CoCo microparticles accelerated limonene release in water and gastric fluids, while decelerating release in intestinal fluid—a result that was opposite of the targeted enteric release. While this particular study did not yield the intended outcomes of slowing volatile release in dry storage and slowing release in acid aqueous media, the resulting data demonstrated the potential of latex excipients in CoCo process for modulating volatile cargo release. For example, formulating with a latex can facilitate applications that desire an initial burst release of the payload from the powder in air or in solution. This study demonstrated the potential of varying excipient levels to control both the initial burst release and long-lasting, sustained release of the payload. Care must be taken, however, that the amount of added latex meet any regulatory requirements for intended use.

## Data availability statement

The original contributions presented in this study are included in the article/supplementary material, further inquiries can be directed to the corresponding author.

## Author contributions

YT: conceptualization, methodology, validation, formal analysis, investigation, writing—original draft and review and editing, visualization, and funding acquisition. HP: investigation. HS: conceptualization and writing—review and editing. TJ: conceptualization, validation, investigation, resources, writing—review and editing, visualization, supervision, project administration, and funding acquisition. All authors contributed to the article and approved the submitted version.

## Abbreviations

CoCo: complex coacervated/complex coacervation
EC: ethylcellulose
pI: isoelectric point
ECC: extent of complex coacervation
SGF: simulated gastric fluid
SIF: simulated intestinal fluid.

## References

[1] Ciriminna R Lomeli-Rodriguez M Demma Carà P Lopez-Sanchez JA Pagliaro M. Limonene: a versatile chemical of the bioeconomy. *Chem Commun.* (2014) 50:15288–96. 10.1039/C4CC06147K 25341412

[2] Arrieta MP López J Hernández A Rayón E. Ternary PLA–PHB–limonene blends intended for biodegradable food packaging applications. *Eur Polym J.* (2014) 50:255–70. 10.1016/j.eurpolymj.2013.11.009

[3] Virot M Tomao V Ginies C Chemat F. Total lipid extraction of food using d-limonene as an alternative to n-hexane. *Chromatographia.* (2008) 68:311–3. 10.1365/s10337-008-0696-1

[4] Hąc-Wydro K Flasiński M Romańczuk K. Essential oils as food eco-preservatives: model system studies on the effect of temperature on limonene antibacterial activity. *Food Chem.* (2017) 235:127–35. 10.1016/j.foodchem.2017.05.051 28554616

[5] John I Muthukumar K Arunagiri A. A review on the potential of citrus waste for D-Limonene, pectin, and bioethanol production. *Int J Green Energy.* (2017) 14:599–612. 10.1080/15435075.2017.1307753

[6] Espina L Gelaw TK de Lamo-Castellví S Pagán R García-Gonzalo D. Mechanism of bacterial inactivation by (+)-Limonene and Its potential use in food preservation combined processes. *PLoS One.* (2013) 8:e56769. 10.1371/journal.pone.0056769 23424676 PMC3570463

[7] Calo JR Crandall PG O’Bryan CA Ricke SC. Essential oils as antimicrobials in food systems – a review. *Food Control.* (2015) 54:111–9. 10.1016/j.foodcont.2014.12.040

[8] d’Alessio PA Ostan R Bisson JF Schulzke JD Ursini MV Béné MC. Oral administration of d-Limonene controls inflammation in rat colitis and displays anti-inflammatory properties as diet supplementation in humans. *Life Sci.* (2013) 92:1151–6. 10.1016/j.lfs.2013.04.013 23665426

[9] Gouin S. Microencapsulation: industrial appraisal of existing technologies and trends. *Trends Food Sci Technol.* (2004) 15:330–47. 10.1016/j.tifs.2003.10.005

[10] Yang Z Peng Z Li J Li S Kong L Li P Development and evaluation of novel flavour microcapsules containing vanilla oil using complex coacervation approach. *Food Chem.* (2014) 145:272–7. 10.1016/j.foodchem.2013.08.074 24128477

[11] Saravanan M Rao KP. Pectin–gelatin and alginate–gelatin complex coacervation for controlled drug delivery: influence of anionic polysaccharides and drugs being encapsulated on physicochemical properties of microcapsules. *Carbohydr Polym.* (2010) 80:808–16. 10.1016/j.carbpol.2009.12.036

[12] Dong Z Ma Y Hayat K Jia C Xia S Zhang X. Morphology and release profile of microcapsules encapsulating peppermint oil by complex coacervation. *J Food Eng.* (2011) 104:455–60. 10.1016/j.jfoodeng.2011.01.011

[13] Tang Y Scher HB Jeoh T. Industrially scalable complex coacervation process to microencapsulate food ingredients. *Innovat Food Sci Emerg Technol.* (2020) 59:102257. 10.1016/j.ifset.2019.102257

[14] Tang Y Scher HB Jeoh T. Volatile retention and enteric release of d-limonene by encapsulation in complex coacervated powders formed by spray drying. *ACS Food Sci Technol.* (2021) 1:2086–95. 10.1021/acsfoodscitech.1c00308

[15] Tang Y. *Microencapsulation of Bioactives by in Situ Complex Coacervation During Spray Drying* Ph.D. Dissertation. Davis, CA: University of Caifornia, Davis (2022).

[16] Tang Y Scher H Jeoh Zicari T. *Microencapsulation of Chemicals and Bioactives by in Situ Complex Coacervation During Spray Drying*. US20210316265A1. Berkeley, CA: University of California (2021).

[17] Tang Y Arbaugh B Park H Scher HB Li B Jeoh T *Targeting Enteric Release of Therapeutic Peptides by Encapsulation in Complex Coacervated Matrix Microparticles by Spray Drying*. Rochester, NY: SSRN (2022). 10.2139/ssrn.4141032

[18] Ahmed AR Mota JP Shahba AAW Irfan M. Chapter 3 - Aqueous polymeric coatings: new opportunities in drug delivery systems. In: Shegokar R editor. *Expectations and Realities of Multifunctional Drug Delivery Systems.* Amsterdam: Elsevier (2020). p. 33–56. 10.1016/B978-0-12-821222-6.00003-8

[19] Petereit HU Weisbrod W. Formulation and process considerations affecting the stability of solid dosage forms formulated with methacrylate copolymers. *Eur J Pharm Biopharm.* (1999) 47:15–25. 10.1016/S0939-6411(98)00083-6 10234523

[20] Lecomte F Siepmann J Walther M MacRae RJ Bodmeier R. Polymer blends used for the aqueous coating of solid dosage forms: importance of the type of plasticizer. *J Control Release.* (2004) 99:1–13. 10.1016/j.jconrel.2004.05.011 15342176

[21] Keddie JL Routh AF. An introduction to latex and the principles of colloidal stability bt - fundamentals of latex film formation. In: Keddie JL Routh AF editors. *Processes and Properties.* Dordrecht: Springer Netherlands (2010). p. 1–26. 10.1007/978-90-481-2845-7_1

[22] Wasilewska K Winnicka K. Ethylcellulose–a pharmaceutical excipient with multidirectional application in drug dosage forms development. *Materials.* (2019) 12:3386. 10.3390/ma12203386 31627271 PMC6829386

[23] Santa-Maria M Scher H Jeoh T. Microencapsulation of bioactives in cross-linked alginate matrices by spray-drying. *J Microencapsulation.* (2012) 29:286–95. 10.3109/02652048.2011.651494 22251237

[24] Jeoh T Wong DE Strobel SA Hudnall K Pereira NR Williams KA How alginate properties influence in situ internal gelation in crosslinked alginate microcapsules (CLAMs) formed by spray drying. *PLoS One.* (2021) 16:e0247171. 10.1371/journal.pone.0247171 33630897 PMC7906420

[25] Strobel SA Scher HB Nitin N Jeoh T. In situ cross-linking of alginate during spray-drying to microencapsulate lipids in powder. *Food Hydrocoll.* (2016) 58:141–9. 10.1016/j.foodhyd.2016.02.031

[26] Swackhamer C Zhang Z Taha AY Bornhorst GM. Fatty acid bioaccessibility and structural breakdown from in vitro digestion of almond particles. *Food Funct.* (2019) 10:5174–87. 10.1039/C9FO00789J 31380548

[27] Chang YI Scire J Jacobs B. *ACS Symposium Series Effect of Particle Size and Microstructure Properties on Encapsulated Orange Oil Flavor Encapsulation.* (Vol. 370). Washington, DC: American Chemical Society (1988). p. 10–87. 10.1021/bk-1988-0370.ch010

[28] Soottitantawat A Yoshii H Furuta T Ohkawara M Linko P. Microencapsulation by spray drying: influence of emulsion size on the retention of volatile compounds. *J Food Sci.* (2003) 68:2256–62. 10.1111/j.1365-2621.2003.tb05756.x

[29] Soottitantawat A Yoshii H Furuta T Ohgawara M Forssell P Partanen R Effect of water activity on the release characteristics and oxidative stability of d-limonene encapsulated by spray drying. *J Agric Food Chem.* (2004) 52:1269–76. 10.1021/jf035226a 14995132

[30] Siepmann J Peppas NA. Modeling of drug release from delivery systems based on hydroxypropyl methylcellulose (HPMC). *Adv Drug Delivery Rev.* (2001) 48:139–57. 10.1016/S0169-409X(01)00112-0 11369079

[31] Jafari SM He Y Bhandari B. Encapsulation of nanoparticles of d-limonene by spray drying: role of emulsifiers and emulsifying techniques. *Null.* (2007) 25:1069–79. 10.1080/07373930701396758

